# The Effect of “Women’s Empowerment” on Child Nutritional Status in Pakistan

**DOI:** 10.3390/ijerph16224499

**Published:** 2019-11-14

**Authors:** Awaisra Shafiq, Abid Hussain, Muhammad Asif, Jinsoo Hwang, Arif Jameel, Shahida Kanwel

**Affiliations:** 1 Department of Economics, Bagdad ul Jadeed Campus, The Islamia University, Bahawalpur 63100, Pakistan; awaisra017@gmail.com; 2 School of Public Affairs, Zijingang Campus, Zhejiang University, Hangzhou 310058, China; abidhusssain02@gmail.com (A.H.); asif.ma015@gmail.com (M.A.); arifjamil24@gmail.com (A.J.); 3 The College of Hospitality and Tourism Management, Sejong University, 98 Gunja-Dong, Gwanjin-Gu, Seoul 143–747, Korea; 4 Hotel & Tourism Management, School of Management, Zhejiang University, Hangzhou 310058, China; shahidakanwel@yahoo.com

**Keywords:** women’s empowerment, child nutrition, the composite index of anthropometric failure (CIAF), socio-economic status, Pakistan

## Abstract

Women’s empowerment in terms of both involvements in employment activities and with decision-making about household activities is the most evident factors that can affect the nutritional and health status of their children. This paper investigates the effect of women’s empowerment (WE) on children’s nutritional (CN) status in Pakistan. The Pakistan demographic health survey (PDHS 2012-13) cross-sectional data was used to analyze the impacts of WE on child malnutrition. The composite index of anthropometric failure (CIAF) was used as a dependent variable to measure the children’s nutritional status, and the wealth index household size. The number of children in a house and indicators of women empowerment, which included the mother’s education, employment status, and the household decision-making, were used as the independent variables. The method of binary logistic regression with marginal effects was used for the empirical analysis of the results. The results of the study showed the indicators of women’s empowerment, such as the education of the mother and employment status had a negative relationship with child malnutrition. Women’s decision-making about the visits to family, which is an indicator of WE, had an insignificant effect on CN. Similarly, socioeconomic status, which included the wealth index, also caused a reduction in child malnutrition. In addition, an increase in household size had a positive and significant relation to child malnutrition. Women are the primary caretakers of children in the household, and their intra-household dynamics affect the well-being of individuals. The empowerment of women acts as a means to enhance children’s nutritional status, which causes important developmental outcomes.

## 1. Introduction

The survival of children, which is due to the exceedance of a proper nutritional status, is influenced by the interactions at the household level. Empowerment plays an important role in most of the aspects of the peoples’ lives [1,2]. In this regard, Chipili et al. [3] argued that the empowerment of women is highly associated with positive child nutritional status. The implications of malnutrition at an early adult age are permanent. It is considered that if malnutrition occurs before a child is two years old or during pregnancy, the result will be a permanent problem regarding the child’s mental and physical development. Women’s empowerment (WE) is an essential indicator to determine a child’s nutritional (CN) status, particularly for children that are less than five years old. Empowering women is also instrumentally valuable to achieve the well-being of men, women, children, and also for developmental issues. Women’s empowerment and the promotion of children’s health were the two main MDGs (Millennium Development Goals) that were set to be achieved by 2015 under the United Nations Development Programmes (UNDP, but the results were unsatisfactory [4]. Women’s empowerment is intangible and latent, and is expressed in several ways, such as mobility, decision-making power, control, and the command of household resources. Economic status and education are considered enabling factors that strongly affect the empowerment of women [5]. 

A previous study described a positive association between WE and involvement in income generating activities and children’s nutritional status [6]. The problem of malnutrition is a multidimensional issue that causes detrimental effects for several reasons. A study in Bangladesh and India concluded that the mother has a great influence on decreasing child stunting if they are well empowered and educated [7]. The enhancement of women’s education is necessary, but it is not a sufficient condition to reduce malnutrition. In developing countries, such as Pakistan, women are educated, but they are not empowered in terms of both household decision-making and income-generating activities. As per the National Nutritional Survey (2011) and the Pakistan Demographic and Health Survey (PDHS) [8], in 2013, malnutrition was prevalent in children under five years old—15% of children were wasting, 44% were stunted, and 31.5% were underweight (Pakistan Economic Survey 2012–13). 

However, the purpose of conducting this research was to measure the associations between women’s empowerment and children’s nutritional status for children less than five years old in Pakistan. Women are considered as the primary caretakers of children in the household, their intra-household dynamics affect individual well-being, and the empowerment of women acts as means to improve the nutritional status of children, which causes important developmental outcomes. The Pakistan demographic health survey (PDHS 2012–13) [8] data were used to analyze the impact of women’s empowerment on child malnutrition through estimation of the composite index of anthropometric failure (CIAF), which shows the significance of this study. 

Researchers have discussed many aspects that determine the relationship between child malnutrition and women’s empowerment. Child health showed a positive relationship with the education of the mother, the age of the mother, income, household size, access to safe drinking water, the location of the house; and a negative relationship between the education of the father, ownership of the assets, the age of the child, and the gender of the child in Ghana [9]). 

Malapit et al. [10] explored the significant relationships between CN and WE, production diversity, individual characteristics, and household characteristics in the rural semi-subsistence households of Nepal. WE reduced the negative impact of low production diversity on children’s diets as well as maternal diets. Similar, a significant connection was found between child nutritional outcomes and women’s empowerment, health care, household purchases, purchases for household needs, visits to relatives, and the husband’s earnings in two developing countries [4]. Significant associations of socioeconomic status and WE with children’s nutrition was estimated in Ethiopia by analyzing the data through Ethiopian demographic and health surveys in 2011 [11]. A positive relationship was found between women’s empowerment and women’s education, the excess of economic resources, and women in the family, and the negative relationship between the dependent variable and decision-making autonomy in a selected rural community in Nigeria [5]. Siddhanta and Chattopadhyay [7] studied the association between WE and child stunting in Eastern Bangladesh and India. The mother’s education and her decision-making power—which are indicators of women’s empowerment—showed a negative and significant relationship with child stunting.

A similar study revealed a significant relationship between WE and CN outcomes in Nigeria and India [12]. An analysis through PDHS [8] found that the mother’s education played a positive role in reducing children’s malnutrition in Malawi, Tanzania, and Zimbabwe [13].

However, the analysis of the previous review determined that women’s empowerment strongly affects child malnutrition. Very few studies exist where CIAF was used to analyze children’s malnutrition, emphasizing the significance of the current study.

## 2. Materials and Methods

### 2.1. Method

The results presented herein are based on the analysis of the 2012-13 Pakistan Demographic and Health Survey [8]. PDHS 2012–13 is the third nationwide survey that was organized under the supervision of the Global Demographic and Health Survey Program. The significance of the survey is that it covers the overall population of the country (see Table 1). For the results analysis, the statistical software STATA was used.

### 2.2. Ethical Considerations

Ethical approval was obtained from the Economics Department of Islamia University Bahawalpur, Punjab, Pakistan (Ref # 69-2017/PREC).

To measure child malnutrition, the composite index of anthropometric failure (CIAF) was taken as a dependent variable and generated by the combination of three anthropometric indices (variables) in the PDHS data set, such as stunting, underweight, and wasting. These indices are expressed according to standard deviation units (Z-score < −2 standard deviation) from the multicenter reference growth study [8] (PDHS report 2012-13); these three measures may not provide an inclusive estimation individually since they can overlap—that is, a child who is stunted could be underweight or wasted or both [14]. However, CIAF is an aggregate measure of the prevalence of malnutrition and incorporates all undernourished children. A child is either stunted, underweight, wasted, or some combination of all three of these as a single variable [15].

- Stunted (low height for age) only;
- Wasted (low weight for height) only;
- Underweight (low weight for age) only;
- Stunted and wasted;
- Stunted and underweight;
- Wasted and underweight;
- Stunted, wasted, and underweight;
- No failure.

The functional form of the model is written as:

*CIAF = β*_0_ + *β*_1_*MEDU + β*_2_*WEMPL + β*_3_*WI + β*_4_*HS* + *β*_5_*DM + ε*_0_.

In binary logistic modeling, the model is explained as

*Y_ij_ = CIAF_ij_ = {*1 *if child is malnourished;* 0 *otherwise}*.

*Y_ij_* is equal to *CIAF_ij_* in binary form—the response of one of only two possible values representing success and failure; or more generally, we can say the presence or absence of an attribute of interest. 

For convenience, only two values were coded as 1 or 0. The distribution of *CIAF_ij_* is a Bernoulli distribution with parameter *πi*, and in compact form it can be written as:

*P_r_ = {CIAF_ij_ = Y_ij_} = πi^Yij^ (*1 – *π_i_)*/ 1 *– Y_ij_*.

For *Y_ij_*, if *Y_ij_ = 1* we obtain *πi*; if *Y_ij_ = 0* we obtain *1 − π_i_* [16].

Multiple logistic regression analysis considers applications involving a single binary dependent variable, here *Y*, and multiple independent variables, denoted *𝑋1, 𝑋2 … 𝑋n*. The form of the multiple linear regression equation is:

*Y_ij_ = ln (**O**) = ln (π_i_*/1 − *π_i_) = β*1 + *β*2*𝑋*1 + *β*3*𝑋*2 +…+ *βn𝑋n* + *ε*_0_,

where “*Y_ij_*“ is binary and represents the CIAF.

In the PDHS, the wealth index is used as a factor to measure the economic and financial status of the household. It is a major household characteristic that is used to measure the socioeconomic status, particularly as it influences health outcome. This index is constructed with three steps using principal component analysis. The household size also affects the distribution of goods and services. In PDHS, an average household size of 6.8 persons was observed. The report indicates the economic pressure that forces families to live together.

The education of the mother is the most important socioeconomic factor that can increase the chances of empowerment at home. In PDHS, the mother’s education is categorized into four parts that help to provide a complete picture of its effects on child nutrition and the empowerment of women. Mothers being better educated helps to empower women and reduces the probability of malnutrition—particularly wasting—among children [17].

Women’s employment is used to measure the development of positive health outcomes. In the PDHS data set, questions related to women’s employment status help to ensure complete coverage in the formal and informal sectors. The variable used in this model is an indicator for women’s empowerment that affects health outcomes of both the children and the mother. Similarly, the variable “household decision-making about visits to family or relatives” is also used to measure the empowerment of women at the household level. This is explained as either woman being empowered with decision making about the household activity, such as health or moving outside the home.

## 3. Results

Frequency, standard deviation and mean values are determined in the form of each variable for individual categories. Frequency shows the distribution of each categorical variables (Composite index of anthropometric failure (CIAF), Mother’s education, Women’s employment, WI (wealth index), and Decision-making about visits to family or relatives) and continuous variables (Household size and No. of children in House) with their mean and value of standard deviation (See Table 2).

The overall mean and standard deviation of each variable without categories shows that household size having highest means value (9.638) with standard deviation (5.276) (see Table 3). 

The values of SD extended from 0.39 to 5.27, and the mean values ranged from 0.52 to 9.63. Pseudo R^2^ and log pseudo likelihood was used to measure the overall effect of CIAF on determinants of women empowerment. The values of pseudo R^2^ (0.0645) and log pseudo likelihood (−1964.8684) indicate that model was significant. The probability values of each variable showed that mother’s education at the secondary and higher level, household size, and wealth index at middle and higher class were significant at the level of 5% (*p* < 0.05). Meanwhile, mothers’ employment and decision-making about health by the husband alone showed significance at the level of 10% (*p* < 0.10). Coefficient values also showed that, as the mother’s education increased the chances of malnutrition decreased. Similarly, mother’s employment status also showed a reduction in the probability of malnutrition. Increase in household size caused an increase in chances of malnutrition. Increase in the wealth index to middle class and rich decreased the probability of malnutrition among children as compared to poor families (see Table 4).

## 4. Discussion

This research investigated and quantified the relationship between women’s empowerment and child nutrition status using data of the 2013 Pakistan Demographic and Health Survey. It is a first attempt in the context of Pakistan to model composite mother‘s empowerment score as one of the determinants of child malnutrition. The results showed that the indicators of women’s empowerment, such as the education of the mother, their employment status, and decision-making about visits to the family by women had a positive and signifcant effect on child nutritional status (i.e., reduced malnutrition). Similarly, higher household wealth status also had a negative and significant effect on CIAF. Household size was positively related to CIAF, indicating higher chances of child malnutrition due to an increase in household size [18]. Malnutrition was found to affect future health outcomes, societal productive potential, and socioeconomic development in Peshawar [18,19].

In Pakistan, economic constraints affect children’s nutritional status by causing child caretakers to save on the cost of fuel by adopting poor food-preparation methods, which impacts the children’s nutritional status [20]. Maternal education is a key indicator to measure children’s nutritional status. Malnutrition is inversely related to the mother’s level of education [18]. Various Pakistani studies have reported that maternal illiteracy is strongly correlated with childhood malnutrition [20,21]. A maternal education that is higher than the primary level is necessary to reduce malnutrition [13].

The mother’s employment status is another important maternal factor to assess malnutrition. If the mother is also an income earner, it helps to increase the total household income, which in turn can increase the chances of obtaining a sufficient quantity of higher-quality food [22]. The mother’s engagement in an unskilled labor occupation positively affected child health in the short term [23]. The relative survival chances of girls increase due to increases in the employment opportunities of adult women [24]. Women’s education and wealth is highly associated with child malnutrition compared to women’s decision-making in the household [25]. As a result, decision makers had little or no association with child malnutrition in this study, which is consistent with the study of Bhagowalia et al. [26].

The results regarding child age indicate that aged children are more likely to suffer from malnutrition than younger children. According to Raju and D’Souza [27] most studies in the context of Pakistan find that age is positively associated with child malnutrition. With respect to the effect of gender on nutrition, the negative sign associated with girls indicates that the likelihood of malnourishment is lower among female children. However, the phenomenon is consistent with general findings in other developing countries where boys naturally have poorer health than girls [28].

An important finding of this research in terms of policy is the positive and highly statistically significant coefficient associated with the variable mother’s education and the high marginal effect of this variable on child malnutrition. The findings support earlier studies on the impact of women’s empowerment regarding health facilities on nutritional status by Alderman and Garcia [29] as well as Headey et al. [30].

## 5. Conclusions

The purpose of the present research was to measure the effects of WE on children’s health and nutritional outcomes. Since it is considered that women are the primary caretakers of children in the household and their intra-household dynamics affect the well-being of individuals, the empowerment of women is as a means to better children’s nutritional status, which influences important developmental outcomes. The Pakistan demographic health survey (PDHS 2012-13) data was used to analyze the impact of women’s empowerment on child malnutrition through the CIAF. The results of the study showed that indicators of women empowerment such as the education of the mother and the mother’s employment status had negative effects on child malnutrition (i.e., malnourishment outcomes decreased). On the other hand, women’s decision-making about the visits to family, an indicator of women’s empowerment, had no significant influence on child nutrition. Similarly, the socioeconomic status (wealth index) also caused a reduction in child malnutrition. Increase in household size had a significant effect promoting child malnutrition.

While the results based on the regression analysis are broadly consistent with what has previously been reported in other studies on malnutrition, they do yield one interesting finding: Women empowerment’s had an edge over child nutritional and household status. Its marginal effect in reducing child malnutrition was larger than estimated for other determinants. This study thus shows that, in the interest of bringing about sustainable improvements in child nutritional status, women’s status in terms of dimensions included in the composite empowerment model should be considered in all interventions by the government of Pakistan, as well as by development partners and international agencies.

## 6. Implications and Future Suggestions

Women’s participation in income-generating activity helps to improve the economic and social status of households, and it also helps to improve the nutritional status of children and reduce gender inequality. So, governmental and non-governmental organizations should establish a formal and informal income-generating sector for women in rural and slum urban areas. 

The mother’s education is essential for a healthy child. Special policies and programs should organize in rural areas and remote urban areas to create awareness about female education. Governments should also actively support programs in both rural and urban areas at the regional and national levels to reduce gender differentials in health, nutrition, education, and employment-generating activities.

## Figures and Tables

**Table 1:** Functional definition of variables.

Variable Name	Functional Definition
Dependent Variable	
CIAF (composite index of anthropometric failure)	1 if child is malnourished, 0 if child is not malnourished
Independent Variable	
MEDU (mother’s education)	0 = No education, 1 = primary, 2 = secondary, 3 = higher
MEMPL (women’s employment)	1 = Yes, 0 = No
WI (wealth index)	1 for poorest, 2 for poor, 3 for middle class, 4 for rich, 5 for richest.
HHS (household size)	Used as continuous variable
DM (household decision-making about visits to family or relatives)	1 for only woman, 2 for both woman and man, 3 for only man

**Table 2:** Frequency analysis of variables.

Variables	Category Percentage	Frequency	Mean	St.d
Composite index of anthropometric failure (CIAF)	Not malnourished (No)	1467	0.477	0.499
	Malnourished (Yes)	1604	0.522	0.499
Mother’s education	No education	6722	0.571	0.494
	Primary education	1687	0.143	0.350
	Secondary education	2077	0.176	0.381
	Higher education	1277	0.108	0.311
Women’s employment	Yes	9483	0.190	0.392
	No	2233	0.809	0.392
WI (wealth index)	Poorest	2758	0.234	0.423
	Poorer	2359	0.200	0.400
	Middle income	2270	0.190	0.394
	Richer	2196	0.186	0.389
	Richest	2180	0.185	0.388
Decision-making about visits to family or relatives	Only woman	761	0.065	0.247
	Man and woman both	3945	0.339	0.473
	Only man	3997	0.334	0.475
Household size	Continuous	1163	9.638	5.276
No. of children in House	Continuous	11763	2.428	1.532

**Table 3:** Descriptive statistics of variables.

Characteristics	Mean	Standard Deviation
CIAF	0.522	0.499
Mother’s education	0.822	1.072
Women’s employment	0.190	0.392
Household size	9.638	5.276
WI (wealth index)	2.88	1.433
Decision-making about visits to family or relatives	2.831	1.110
No. of children in House	2.428	1.532

**Table 4:** Binary logistic results for CIAF.

Variables	Coefficients	Marginal Effects (dy/dx)
	Mother’s education (No education, reference category)	
Primary	−0.071	−0.013
Secondary	−0.692	−0.147
Higher	−0.633	−0.126
	Mothers employment (Not employed, reference category)	
Employed	−0.167	−0.02
	Household size	
	0.028	0.001
	Decision-making about visits to family or relatives(Woman alone, reference category)	
Woman and husband both	−0.148	−0.031
Husband alone	−0.302	−0.058
Family elders	−0.263	−0.057
	Wealth index (Poorest as a reference category)	
Poor	−0.175	−0.035
Middle class	−0.456	−0.076
Rich	−0.674	−0.128
	No. of children in house (Continuous variable)	
	0.013	0.0015
	Overall significance of the model	
Number of observations		3035
Pseudo R2		0.0645
Log likelihood		−1964.8684
